# Association of Cholecystectomy With Liver Fibrosis and Cirrhosis Among Adults in the USA: A Population-Based Propensity Score-Matched Study

**DOI:** 10.3389/fmed.2021.787777

**Published:** 2021-11-30

**Authors:** Zhi-Qin Xie, Hong-Xia Li, Wen-Liang Tan, Lei Yang, Xiao-Wu Ma, Wen-Xin Li, Qing-Bin Wang, Chang-Zhen Shang, Ya-Jin Chen

**Affiliations:** ^1^Department of Hepatobiliary Surgery, Sun Yat-sen Memorial Hospital, Sun Yat-sen University, Guangzhou, China; ^2^Department of Pathology, Zhuzhou Hospital Affiliated to Xiangya School of Medicine, Central South University, Zhuzhou, China; ^3^Department of Cardiology, The Eighth Affiliated Hospital, Sun Yat-sen University, Shenzhen, China

**Keywords:** cholecystectomy, non-alcoholic fatty liver disease, liver fibrosis, liver cirrhosis, association

## Abstract

**Background and Aims:** Cholecystectomy is the “gold standard” for treating diseases of the gallbladder. In addition, non-alcoholic fatty liver disease (NAFLD), liver fibrosis or cirrhosis, are major causes of morbidity and mortality across the world. However, the association between cholecystectomy and these diseases is still unclear. We assessed the association among US adults and examined the possible risk factors.

**Methods:** This cross-sectional study used data from 2017 to 2018 National Health and Nutrition Examination Survey, a population-based nationally representative sample of US. Liver fibrosis and cirrhosis were defined by median stiffness, which was assessed by transient elastography. Furthermore, patients who had undergone cholecystectomy were identified based on the questionnaire. In addition, Propensity Score Matching (PSM, 1:1) was performed based on gender, age, body mass index (BMI) and diabetes.

**Results:** Of the 4,497 included participants, cholecystectomy was associated with 60.0% higher risk of liver fibrosis (OR:1.600;95% CI:1.278–2.002), and 73.3% higher risk of liver cirrhosis (OR:1.733, 95% CI:1.076–2.792). After PSM based on age, gender, BMI group and history of diabetes, cholecystectomy was associated with 139.3% higher risk of liver fibrosis (OR: 2.393;95% CI: 1.738–3.297), and 228.7% higher risk of liver cirrhosis (OR: 3.287, 95% CI: 1.496–7.218).

**Conclusions:** The present study showed that cholecystectomy is positively associated with liver fibrosis and cirrhosis in US adults. The discovery of these risk factors therefore provides new insights on the prevention of NAFLD, liver fibrosis, and cirrhosis.

## Introduction

Chronic liver diseases, such as non-alcoholic fatty liver disease (NAFLD), liver fibrosis (LF) or cirrhosis (LC), are major causes of morbidity and mortality across the world (1–3). With the rising prevalence of NAFLD, interest is increasing in LF, which is a reversible condition and can progress to irreversible LC and even hepatocellular carcinoma (HCC), thereby leading to a major social and economic burden (4, 5). Previous researches have reported that LF is correlated with long-term outcomes of NAFLD patients (6). However, liver fibrosis continues to threaten public health despite decades of research, and thus scientists are now focused on prevention strategies. Classically, LF is caused by various risk factors, such as viral hepatitis, alcoholism, obesity, and type 2 diabetes (4). Better understanding of the associated risk factors may contribute to the early prevention of the underlying liver disease.

Gallbladder diseases are also among the most prevalent conditions worldwide, affecting 10 to 20% of the adult population (7). Cholecystectomy is widely used as the “gold standard” for the treatment of gallbladder diseases, such as gallstones, acute cholecystitis and benign tumors of the gallbladder (8). However, few studies have evaluated whether cholecystectomy is associated with an increased risk of developing NAFLD. A previous retrospective, multicenter study in Turkey showed that there is no independent association between the presence of cholecystectomy and advanced LF (9). The study focused on whether the presence of gallstones in patients with biopsy-proven NAFLD was associated with advanced LF and histological non-alcoholic steatohepatitis (NASH), providing preliminary reference for research on hepatology and cholecystectomy. Unfortunately, the main shortcoming of the study was the small sample size which included 41 cases of cholecystectomy and 387 without. On the contrary, another cross-section study using data of the third US National Health and Nutrition Examination Survey (NHANES III, 1988–1994), showed the positive association between NAFLD and cholecystectomy (10). Moreover, no further study on the association between cholecystectomy and LF or LC has been conducted ever since. Recently, ultrasound transient elastography (TE) was widely used to evaluate liver fibrosis in chronic liver diseases in a non-invasive and reproducible manner (11). Notably, transient elastography was first conducted in the NHANES 2017–2018 cycle, providing opportunity to assess the weak connection between cholecystectomy and LF or LC.

Consequently, the present study sought to examine the association between cholecystectomy and LF or LC using a nationally representative sample of US adults from the NHANES 2017–2018.

## Materials and Methods

### Study Population

This study analyzed data from NHANES 2017–2018, where ultrasound TE of the liver was first conducted. The NHANES was a national, cross-sectional survey that assessed the health and nutritional status of individuals in the United States. A detailed description of NHANES has been published elsewhere (12). During the 2017–2018 cycle of NHANES, 9,254 participants finished the survey. However, the present study excluded individuals who were <20 years old and could not undergo TE (*N* = 4,744). Patients with autoimmune hepatitis and those lacking data of cholecystectomy were also excluded from further analysis (*N* = 13). Consequently, 4,497 participants were enrolled for further analysis. Moreover, written informed consent was obtained from all the participants and the survey protocol was approved by the Research Ethics Review Board of the National Center for Health Statistics. Additionally, specific informed consent was not required for this secondary analysis of the publicly available data. This report was also drafted according to the reporting guidelines for cross-sectional studies, stipulated by Strengthening the Reporting of Observational Studies in Epidemiology (STROBE) (13).

### Primary Exposure

Patients who had undergone cholecystectomy were identified based on self-reports and this information was acquired from the “medical conditions” section of the questionnaire. In addition, 8,897 participants who were older than 20 years answered the question; “Ever had cholecystectomy?” Notably, 641 (7.2%) individuals answered “Yes” while 4,925 (55.4%) participants answered “No”.

### Outcomes

During NHANES 2017–2018, TE was first conducted by educated health technicians. Additionally, liver stiffness was measured using the FibroScan® model 502 V2 Touch, which used ultrasound and vibration-controlled TE. Notably, TE is a widely used, noninvasive and reliable method of evaluating LF or LC (14, 15). All participants older than 12 years of age were eligible except for individuals who could not lie on the exam table, had an implanted electronic medical device, were pregnant or had a lesion at the site of examination. In addition, only individuals with complete tests (a fasting time of 3 hours, complete stiffness ≥10 measures and interquartile range of liver stiffness/median stiffness <30%) were enrolled in this study. Moreover, LF was defined as F0-F4, with the cutoff values of median liver stiffness (LSM) being 6.3, 8.3, 10.5 and 12.5 (KPa), respectively (16). Furthermore, Significant LF and LC was defined as LSM ≥ 6.3 KPa (fibrosis grade ≥ F1) and LSM ≥ 12.5 KPa (fibrosis grade ≥ F4), respectively (16, 17).

### Covariates

Covariates were selected based on known confounders from previous literature and clinical practice. Briefly, demographic factors such as age, sex and race/ethnicity were included first. In addition, levels of education, alcohol use, diabetes, HBV infection, HCV infection, physical activity status, serum cotinine levels, Body Mass Index (BMI), and the poverty income ratio were also evaluated through interviews.

In this study, age was classified into six categories: 20–29, 30–39, 40–49, 50–59, 60–69, and 70–80 years. In NHANES 2017–2018, race/ethnicity was classified as Hispanic (referring to all Hispanics), non-Hispanic White (referring to whites with no Hispanic origin), non-Hispanic Black (meaning blacks with no Hispanic origin), non-Hispanic Asian (meaning Asians with no Hispanic origin) or other races including Alaska Natives or American Indians, Native Hawaiians or other Pacific Islanders and multiracial individuals. In addition, the BMI was categorized into three groups: under/normal weight (<25.0 kg/m^2^), overweight (25.0–29.9 kg/m^2^ and obesity (≥30.0 kg/m^2^). Participants with diabetes were also defined as those with a self-reported history of diagnosis with diabetes or glycohemoglobin ≥ 6.5% (18). Moreover, individuals with HCV or HBV infections were identified based on positive diagnostic tests (19, 20) or self-reported infection.

Furthermore, current alcohol use was categorized as none, moderate (>0 to ≤ 2 drinks/d for men or >0 to ≤ 1 drink/d for women), heavy (>2 to <5 drinks/d for men or >1 to <4 drink/d for women) or binge (≥5 drinks/d for men or ≥4 drink/d for women) based on recommendations from the National Institute on Alcohol Abuse and Alcoholism (NIAAA) in the National Institute of Health. On the other hand, smoking was categorized according to the serum cotinine levels into low (<0.015 ng/ml), moderate (0.015–3 ng/ml) and high levels (>3 ng/ml) (21). Moreover, the participants were categorized into three groups: active (≥the recommended level of activity), less active (< the recommended level of activity) and inactive (no activity), based on evidence that more than 75 min of vigorous or 150 min of moderate physical activity per week is recommended for Americans (22). In addition, the level of income was measured using the poverty income ratio (ratio of family income to poverty threshold) and was classified into three categories: <1.3, 1.3–1.8, and >1.8. The level of education (more than high school education, high school education, less than high school education) and laboratory-measured levels of Alanine Aminotransferase (ALT), Aspartate Aminotransferase (AST), Albumin (ALB), Alkaline Phosphatase (ALP), γ-glutamyl Transpeptidase (GGT), Total Cholesterol (TC), Total Bilirubin (TB) and platelet were also evaluated through interviews.

### Statistical Analysis

Continuous variables were expressed as the weighted mean (standard deviation) and comparisons between two groups were made using the independent samples *T*-test or Mann-Whitney test. In addition, categorical variables were described by weighted percentages (95% confidence interval, 95% CI) and compared using the χ^2^ test. Multivariate logistic regression analysis was also performed to evaluate the correlation between LF, LC and cholecystectomy. The final model was adjusted for age, gender, race, level of education, alcohol use, diabetes, HBV infection, HCV infection, physical activity status, serum cotinine levels, BMI, and the poverty income ratio.

Additionally, subgroup analyses were conducted by examining age, gender, race/ethnicity. Propensity Score Matching (PSM) was also applied to match two groups, with a ratio of 1:1 and a clipper of 0.00 using SPSS version 25.0 (IBM, New York, USA).

All the statistical analysis were performed using the R software (http://www.R-project.org, The R Foundation) and Empowerstats (http://www.empowerstats.com, X&Y Solutions, Inc), with appropriate interview/examination weights to represent the complex survey design. Moreover, 2-sided tests were used to obtain all the *p* values and statistical significance was set at *p* < 0.05.

## Results

### Overall Characteristics of the Participants

**Figure 1:**
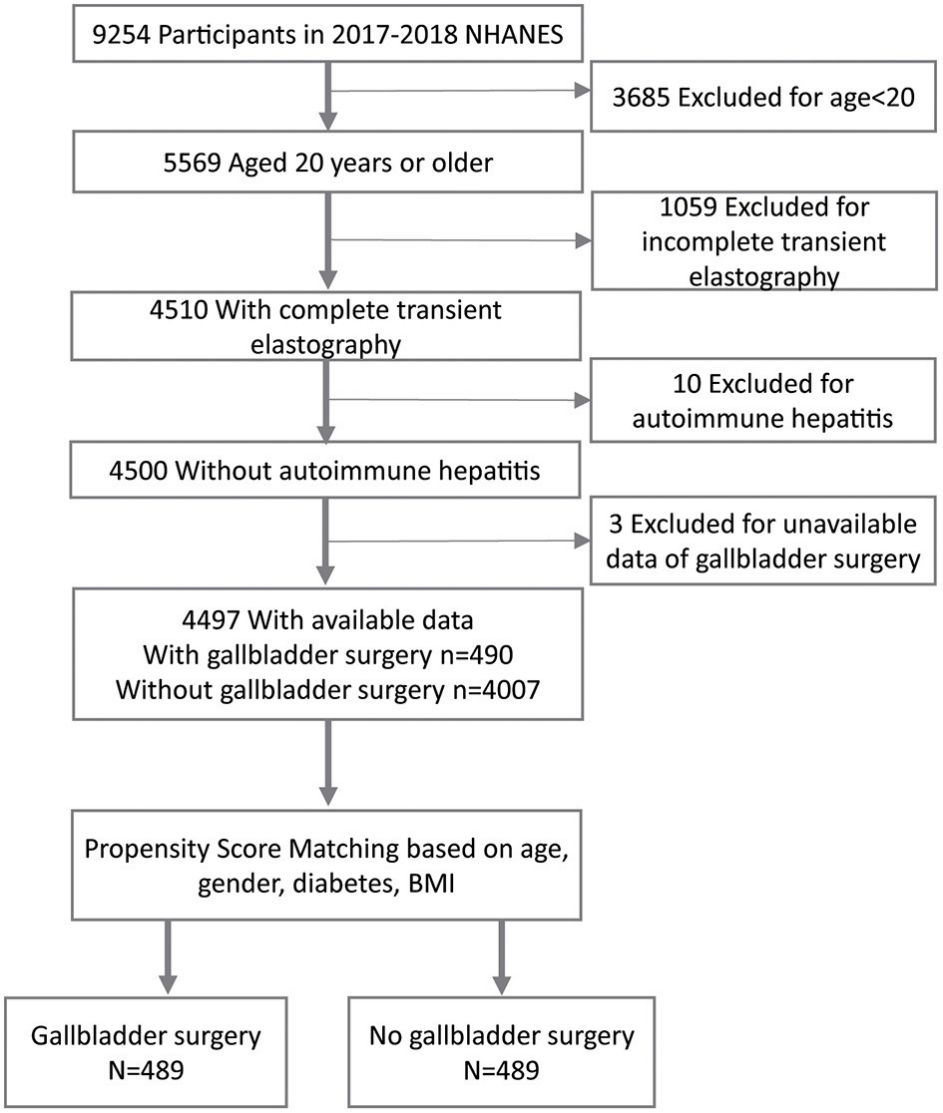
A flowchart showing the selection of study participants. BMI-Body Mass Index.

A total of 4,497 participants who were older than 20 years in NHANES 2017–2018, were included in this analysis. Herein, 490 individuals had undergone cholecystectomy while 4,007 participants had not (Figure 1). The overall characteristics of the included participants are shown in Table 1. Table 1 showed that individuals who had undergone cholecystectomy were mostly older (57.21 ± 14.62 years vs. 46.68 ± 16.95 years, *p* < 0.001), female [77.3% (95% CI, 73.6–81.0%) vs. 47.1% (95% CI, 45.6–48.6%), *p* < 0.001], non-Hispanic Whites [73.2% (95% CI, 69.3–77.1%) vs. 61.1% (95% CI, 59.6–62.6%), *p* < 0.001], obese [57.8% (95% CI, 53.4–62.2%) vs. 38.8% (95% CI, 37.3–40.3%), *p* < 0.001], had less physical activity levels (12.5 vs. 7.1%, *p* < 0.001), had diabetes [25.0% (95% CI, 21.2–28.8%) vs. 11.3% (95% CI, 10.3–12.3%), *p* < 0.001] and had lower serum cotinine levels [less than 0.015 ng/mL, 46.5% (95% CI, 42.1–50.9%) vs. 35.7% (95% CI, 34.2–37.2%), *p* < 0.001], compared to those who has not received the surgery. Moreover, participants who had undergone cholecystectomy had higher levels of ALP (82.89 ± 33.52 U/L vs. 75.44 ± 22.99 U/L, *p* < 0.001), platelets (255.77 ± 71.75 × 10^9^/L vs. 243.21 ± 58.68 × 10^9^/L, *p* < 0.001), median liver stiffness (6.71 ± 6.45 KPa vs. 5.52 ± 4.27 KPa, *p* < 0.001), controlled attenuated parameter (279.84 ± 59.85 dB/m vs. 261.35 ± 62.53 dB/m, *p* < 0.001), than those who had not received the surgery.

**Table 1:** General characteristics of included participants (*n* = 4497) by the presence or absence of a history of cholecystectomy in the NHANES 2017–2018.

**Characters**	**Yes** **(*n* = 490)**	**No** **(*n* = 4007)**	***p*-Value**
Age (years)	57.21 ± 14.62	46.68 ± 16.95	<0.001
20–29	4.3 (2.5–6.1)	20.6 (19.3–21.9)	
30–39	10.6 (7.9–13.3)	19.1 (17.9–20.3)	
40–49	17.0 (13.7–20.3)	15.6 (14.5–16.7)	
50–59	18.9 (15.4–22.4)	19.2 (18.0–20.4)	
60–69	27.5 (23.6–31.5)	14.6 (13.5–15.7)	
70–80	21.8 (18.1–25.5)	10.9 (9.9–11.9)	
Gender			<0.001
Male	22.7 (19.0–26.4)	52.9 (51.4–54.4)	
Female	77.3 (73.6–81.0)	47.1 (45.6–48.6)	
Race/ethnicity			<0.001
Hispanic	11.7 (8.9–14.6)	16.3 (15.2–17.4)	
Non-Hispanic White	73.2 (69.3–77.1)	61.1 (59.6–62.6)	
Non-Hispanic Black	6.5 (4.3–8.7)	11.8 (10.8–12.8)	
Non-Hispanic Asian	2.2 (0.9–3.5)	6.3 (5.5–7.1)	
Other races^a^	6.4 (4.2–8.6)	4.5 (3.9–5.1)	
Education			0.184
More than high school	58.3 (53.9–62.7)	62.6 (61.1–64.1)	
High school or equivalent	30.4 (26.3–34.5)	26.3 (24.9–27.7)	
Less than high school	11.3 (8.5–14.1)	11.0 (10.0–12.0)	
Not recorded	0.0	0.1 (0.0–0.2)	
Poverty-income ratio			0.928
<1.3	17.1 (13.8–20.4)	17.6 (16.4–18.8)	
1.3–1.8	8.4 (5.9–10.9)	8.2 (7.4–9.0)	
>1.8	65.0 (60.8–69.2)	63.9 (62.4–65.4)	
Not recorded	9.5 (6.9–12.1)	10.3 (9.4–11.2)	
BMI group			<0.001
<25	13.8 (10.8–16.9)	28.9 (27.5–30.3)	
25–30	27.9 (23.9–31.9)	31.8 (30.4–33.2)	
≥30	57.8 (53.4–62.2)	38.8 (37.3–40.3)	
Not recorded	0.6 (−0.1, 1.3)	0.6 (0.4–0.8)	
Physical activity level			<0.001
Inactive	51.2 (46.8–55.6)	50.8 (49.3–52.3)	
Less active	12.5 (9.6–15.4)	7.1 (6.3–7.9)	
Active	36.4 (32.1–40.7)	42.1 (40.6–43.6)	
Daily alcohol drinking status			<0.001
Non-drinkers	7.4 (5.1–9.7)	7.1 (6.3–7.9)	
Moderate-drinkers	30.5 (26.4–34.6)	29.6 (28.2–31.0)	
Heavy-drinkers	17.8 (14.4–21.2)	13.6 (12.5–14.7)	
Binge-drinkers	22.0 (18.3–25.7)	34.4 (32.9–35.9)	
Not recorded	22.3 (18.6–26.0)	15.3 (14.2–16.4)	
History of diabetes			<0.001
Yes	25.0 (21.2–28.8)	11.3 (10.3–12.3)	
Having HBV infection			0.009
Yes	1.0 (0.1–1.9)	0.9 (0.6–1.2)	
Having HCV infection			0.006
Yes	2.9 (1.4–4.4)	2.4 (1.9–2.9)	
**Laboratory parameters**			
Smoking (serum cotinine levels)			<0.001
<0.015	46.5 (42.1–50.9)	35.7 (34.2–37.2)	
0.015–3	29.5 (25.5–33.5)	36.7 (35.2–38.2)	
≥3	22.6 (18.9–26.3)	23.5 (22.2–24.8)	
Not recorded	1.4 (0.4–2.4)	4.1 (3.5–4.7)	
ALT (U/L)	20.98 ± 14.97	23.65 ± 17.13	<0.001
AST (U/L)	20.15 ± 10.31	22.58 ± 13.29	<0.001
ALP (U/L)	82.89 ± 33.52	75.44 ± 22.99	<0.001
ALB (g/L)	39.68 ± 3.23	41.13 ± 3.06	<0.001
GGT (U/L)	30.79 ± 62.64	30.10 ± 35.28	0.706
TC (mmol/L)	4.83 ± 0.94	4.91 ± 1.03	0.098
TB (umol/L)	7.99 ± 4.87	8.09 ± 4.60	0.632
Platelet (×10^9^/L)	255.77 ± 71.75	243.21 ± 58.68	<0.001
Transient Elastography			
Median stiffness (kPa)	6.71 ± 6.45	5.52 ± 4.27	<0.001
Controlled attenuated parameter (dB/m)	279.84 ± 59.85	261.35 ± 62.53	<0.001
Liver fibrosis status			<0.001
Yes	34.7(30.5–38.9)	19.8(18.6–21.0)	
Liver cirrhosis			0.041
Yes	4.5 (2.7–6.3)	2.8 (2.3–3.3)	

*Values are weighted mean ± SD or weighted % (95% confidence interval). P values are weighted*.

a *Other races include American Indian or Alaska Native, Native Hawaiian or other Pacific Islander, and multiracial persons*.

*NHANES, National Health and Nutrition Examination Survey; BMI, body mass index; HBV, hepatitis B virus; HCV, hepatitis C virus; ALT, alanine aminotransferase; AST, aspartate aminotransferase; ALP, alkaline Phosphatase; ALB, albumin; GGT, gamma glutamyl transferase; TC, total cholesterol; TB, total bilirubin*.

Moreover, the incidence of LF and LC was higher in participants who had received cholecystectomy [34.7% (95% CI, 30.5–38.9%) vs. 19.8% (95% CI, 18.6–21.0%), *p* < 0.001; 4.5% (95% CI, 2.7–6.3%) vs. 2.8% (95% CI, 2.3–3.3%), *p* = 0.041, respectively].

### Characteristics of Participants After PSM

Given the significant differences at baseline between included participants who had undergone cholecystectomy and those who had not, PSM was performed on the individuals using such covariates as age, gender, BMI group and history of diabetes, which were previously associated with metabolic disorder and liver disease (23–25). After PSM, 489 pairs of cases were further analyzed (Table 2). The findings showed that gender, age, level of education, diabetes, HBV infection, HCV infection, serum cotinine levels, BMI group, and the poverty income ratio, were comparable between the two groups after PSM. Compared to participants who had not received cholecystectomy after PSM, more individuals who had undergone cholecystectomy were Non-Hispanic Whites [73.2% (95% CI, 69.3–77.1%) vs. 59.9% (95% CI, 55.6–64.2%), *p* < 0.001], heavy-drinkers [17.8% (95% CI, 14.4–21.2%) vs. 14.3% (95% CI, 11.2–17.4%), *p* = 0.006], had higher levels of physical activity (less active: 12.5 vs. 7.7%; active: 36.3 vs. 32.6%; *p* = 0.009), TB (7.99 ± 4.93 umol/L vs. 7.14 ± 4.45 umol/L, *p* = 0.007), platelet [(255.86 ± 72.11) × 10^9^/L vs. (245.84 ± 60.77) × 10^9^/L, *p* = 0.023), and higher values of median stiffness (6.71 ± 6.46 KPa vs. 5.03 ± 2.41 KPa, *p* < 0.001) as well as controlled attenuated parameter (279.75 ± 59.85 dB/m vs. 266.38 ± 58.55, *p* < 0.001). However, those who had undergone cholecystectomy had lower levels of ALB (39.66 ± 3.27 g/L vs. 40.46 ± 2.94 g/L, *p* < 0.001), TC (4.83 ± 0.95 mmol/L vs. 5.11 ± 1.22 mmol/L, *p* < 0.001) and AST (20.10 ± 10.45 U/L vs. 21.72 ± 12.15 U/L, *p* = 0.030). Moreover, the incidence of LF (≥F1) was more than two-fold higher in participants who had received cholecystectomy [34.6% (95% CI, 30.4–38.8%) vs. 16.4% (95% CI, 13.1–19.7%), *p* < 0.001), and LC (≥F4) was threefold higher [4.5% (95% CI, 2.7–6.3%) vs. 1.5% (95% CI, 0.4–2.6%), *p* = 0.0099].

**Table 2:** General characteristics of participants (*n* = 978) by the presence or absence of a history of cholecystectomy after propensity score matching in the NHANES 2017–2018.

**Characters**	**Yes** **(*n* = 489)**	**No** **(*n* = 489)**	***p*-Value**
Age (years)	57.23 ± 14.61	56.76 ± 15.09	0.624
20–29	4.3 (2.5–6.1)	4.5 (2.7–6.3)	
30–39	10.6 (7.9–13.3)	8.9 (6.4–11.4)	
40–49	16.9 (13.6–20.2)	16.2 (12.9–19.5)	
50–59	18.9 (15.4–22.4)	18.6 (15.2–22.0)	
60–69	27.6(23.6–31.6)	21.9 (18.2–25.6)	
70–80	21.9 (18.2–25.6)	29.9 (25.8–34.0)	
Gender			0.343
Male	22.7 (19.0–26.4)	25.3 (21.4–29.2)	
Female	77.3 (73.6–81.0)	74.7 (70.8–78.6)	
Race/ethnicity			<0.001
Hispanic	11.7 (8.9–14.5)	17.0 (13.7–20.3)	
Non-Hispanic White	73.2 (69.3–77.1)	59.9 (55.6–64.2)	
Non-Hispanic Black	6.5 (4.3–8.7)	12.0 (9.1–14.9)	
Non-Hispanic Asian	2.2 (0.9–3.5)	6.4 (4.2–8.6)	
Other races^a^	6.5 (4.3–8.7)	4.8 (2.9–6.7)	
Education			0.086
More than high school	58.2 (53.8–62.6)	60.9 (56.6–65.2)	
High school or equivalent	30.5 (26.4–34.6)	24.1 (20.3–27.9)	
Less than high school	11.3 (8.5–14.1)	14.8 (11.7–17.9)	
Not recorded	0.0	0.1 (−0.2, 0.4)	
Poverty-income ratio			0.988
<1.3	17.2 (13.9–20.5)	17.2 (13.9–20.5)	
1.3–1.8	8.2 (5.8–10.6)	8.3 (5.9–10.7)	
>1.8	65.1 (60.9–69.3)	65.7 (61.5–69.9)	
Not recorded	9.5 (6.9–12.1)	8.8 (6.3–11.3)	
BMI group			0.417
<25	13.8 (10.7–16.9)	13.7 (10.7–16.7)	
25–30	28.5 (24.5–32.5)	24.9 (21.1–28.7)	
≥30	57.7 (53.3–62.1)	61.5 (57.2–65.8)	
Physical activity level			0.009
Inactive	51.3 (46.9–55.7)	59.8 (55.5–64.1)	
Less active	12.5 (9.6–15.4)	7.7 (5.3–10.1)	
Active	36.3 (32.0–40.6)	32.6 (28.4–36.8)	
Daily alcohol drinking status			0.006
Non-drinkers	7.4 (5.1–9.7)	6.4 (4.2–8.6)	
Moderate-drinkers	30.4 (26.3–34.5)	36.0 (31.7–40.3)	
Heavy-drinkers	17.8 (14.4–21.2)	14.3 (11.2–17.4)	
Binge-drinkers	22.1 (18.4–25.8)	28.2 (24.2–32.2)	
Not recorded	22.3 (18.6–26.0)	15.1 (11.9–18.3)	
History of diabetes			0.900
Yes	24.9 (21.1–28.7)	25.1 (21.3–28.9)	
Having HBV infection			0.128
Yes	1.0 (0.1–1.9)	1.4 (0.4–2.4)	
Having HCV infection			0.053
Yes	2.9 (1.4–4.4)	2.1 (0.8–3.4)	
**Laboratory parameters**			
Smoking (serum cotinine levels)			0.183
<0.015	46.5 (42.1–50.9)	44.4 (40.0–48.8)	
0.015–3	29.6 (25.6–33.6)	33.5 (29.3–37.7)	
≥3	22.5 (18.8–26.2)	19.3 (15.8–22.8)	
Not recorded	1.4 (0.4–2.4)	2.8 (1.3–4.3)	
ALT (U/L)	20.92 ± 15.17	21.92 ± 14.52	0.314
AST (U/L)	20.10 ± 10.45	21.72 ± 12.15	0.030
ALP (U/L)	83.01 ± 33.97	80.50 ± 25.16	0.216
ALB (g/L)	39.66 ± 3.27	40.46 ± 2.94	<0.001
GGT (U/L)	30.67 ± 63.47	31.63 ± 41.87	0.794
TC (mmol/L)	4.83 ± 0.95	5.11 ± 1.22	<0.001
TB (umol/L)	7.99 ± 4.93	7.14 ± 4.45	0.007
Platelet (×10^9^/L)	255.86 ± 72.11	245.84 ± 60.77	0.023
Transient Elastography			
Median stiffness (kPa)	6.71 ± 6.46	5.03 ± 2.41	<0.001
Controlled attenuated parameter (dB/m)	279.75 ± 59.85	266.38 ± 58.55	<0.001
Liver fibrosis			<0.001
Yes	34.6 (30.4–38.8)	16.4 (13.1–19.7)	
Liver cirrhosis			0.0099
Yes	4.5 (2.7–6.3)	1.5 (0.4–2.6)	

*Values are weighted mean ± SD or weighted % (95% confidence interval). p values are weighted*.

a *Other races include American Indian or Alaska Native, Native Hawaiian or other Pacific Islander, and multiracial persons*.

*NHANES, National Health and Nutrition Examination Survey; BMI, body mass index; HBV, hepatitis B virus; HCV, hepatitis C virus; ALT, alanine aminotransferase; AST, aspartate aminotransferase; ALP, alkaline Phosphatase; ALB, albumin; GGT, gamma glutamyl transferase; TC, total cholesterol; TB, total bilirubin; NAFLD, non-alcoholic fatty liver disease*.

### Associations Between Cholecystectomy and LF

After PSM and unadjusted analysis (Table 3), the OR value for the presence of LF in participants who had undergone cholecystectomy was 2.130 (95% CI, 1.598–2.839), compared to those who had not received the surgery. This value remained statistically significant after adjusting for gender, age, and race (OR, 2.149 [95% CI, 1.598–2.891]). In addition, there was an increase in the OR value for the association of LF with cholecystectomy, after full adjustment (2.393 [95% CI, 1.738–3.297]).

**Table 3:** Associations between cholecystectomy and liver fibrosis after propensity score matching (*n* = 978), NHANES 2017–2018.

	**Model 1 OR (95% CI),** **P**	**Model 2 OR (95% CI),** **P**	**Model 3 OR (95% CI),** **P**
Cholecystectomy			
No	Reference	Reference	Reference
Yes	2.130 (1.598, 2.839) <0.001	2.149 (1.598, 2.891) <0.001	2.393 (1.738, 3.297) <0.001
Stratified by age			
20–29y	Inf. (0.000, Inf)^*^ 0.996	Inf. (0.000, Inf)0.997	105266.561 (0.000, Inf) 1.000
30–39y	4.154 (1.341, 12.870) 0.014	3.016 (0.869, 10.465) 0.082	1.134 (0.152, 8.466) 0.903
40–49y	2.375 (1.108, 5.092) 0.026	2.563 (1.155, 5.686) 0.021	4.287 (1.407, 13.060) 0.010
50–59y	3.804 (1.910, 7.576) <0.001	4.380 (2.110, 9.090) <0.001	5.488 (2.238, 13.461) <0.001
60–69y	1.384 (0.790, 2.426) 0.256	1.435 (0.807, 2.553) 0.219	1.728 (0.847, 3.523) 0.132
70–80y	1.600 (0.959, 2.669) 0.072	1.590 (0.934, 2.705) 0.087	1.585 (0.866, 2.901) 0.135
Stratified by gender			
Men	1.810 (1.073, 3.054) 0.026	1.972 (1.130, 3.441) 0.017	2.153 (1.113, 4.166) 0.023
Women	2.309 (1.631, 3.268) <0.001	2.247 (1.572, 3.211) <0.001	2.555 (1.727, 3.778) <0.001
Stratified by race			
Hispanic	1.832 (1.042, 3.218) 0.035	1.751 (0.978, 3.135) 0.060	1.496 (0.736, 3.038) 0.265
Non-Hispanic White	3.253 (1.959, 5.404) <0.001	3.199 (1.918, 5.334) <0.001	3.835 (2.186, 6.730) <0.001
Non-Hispanic Black	1.023 (0.529, 1.978) 0.947	1.038 (0.518, 2.079) 0.916	1.276 (0.559, 2.912) 0.562
Non-Hispanic Asian	2.700 (1.004, 7.263) 0.049	2.174 (0.749, 6.310) 0.153	5.924 (0.912, 38.490) 0.062
Other races	2.422 (0.753, 7.786) 0.138	3.671 (0.946, 14.247) 0.060	106.704 (1.434, 7938.920) 0.034

*Model 1: Non-adjusted model; Model 2 adjusted for: gender; age; race; Model 3 adjusted for: gender; age; race; education; alcohol; diabetes; HBV infection; HCV infection; physical activity status; serum cotinine levels; BMI, and poverty income ratio*.

* *‘Inf' means that values can't be calculated*.

*NHANES, National Health and Nutrition Examination Survey; BMI, body mass index; HBV, hepatitis B virus; HCV, hepatitis C virus*.

In addition, subgroup analyses revealed that cholecystectomy patients who are 40–49 years old, 50–59 years old, female, or Non-Hispanic White are at a higher risk of developing LF regardless of whether PSM was performed. After PSM, the OR value for the association of LF with cholecystectomy remained significant in participants who were 40–49 years old (Full adjustment: 4.287 [95% CI, 1.407–13.060]) and 50–59 years of age (Full adjustment: 5.488 [95% CI, 2.238–13.461]). After stratification by gender, the OR value remained significant especially in females (Full adjustment: 2.555 [95% CI, 1.727–3.778]). Additionally, there were significant associations between cholecystectomy and other covariates, including non-Hispanic Whites (Full adjustment: OR, 3.835 [95% CI, 2.186–6.730]).

### Associations Between Cholecystectomy and LC

Finally, the study assessed the association between cholecystectomy and LC (Table 4) in participants after PSM. After PSM and unadjusted analysis, the OR value for the presence of LC in participants who had undergone cholecystectomy was 3.020 (95% CI, 1.455–6.267), compared to those who had not received the surgery. This value remained statistically significant after adjusting for gender, age, and race (OR, 3.030 [95% CI, 1.435–6.395]). In addition, there was an increase in the OR value for the association of LC with cholecystectomy, after full adjustment (3.287 [95% CI, 1.496–7.218]).

**Table 4:** Associations between cholecystectomy and liver cirrhosis after propensity score matching (*n* = 978), NHANES 2017–2018.

	**Model 1 OR (95% CI),** **P**	**Model 2 OR (95% CI),** **P**	**Model 3 OR (95% CI),** **P**
Cholecystectomy			
No	Reference	Reference	Reference
Yes	3.020 (1.455, 6.267) 0.003	3.030 (1.435, 6.395) 0.004	3.287 (1.496, 7.218) 0.003
Stratified by Age			
20–29y	1.000 (0.000, Inf)^*^ 1.000	1.000 (0.000, Inf) 1.000	1.000 (0.000, Inf) 1.000
30–39y	Inf. (0.000, Inf) 0.995	Inf. (0.000, Inf)0.998	Inf. (0.000, Inf) 1.000
40–49y	Inf. (0.000, Inf) 0.996	Inf. (0.000, Inf)0.999	123814.773 (0.000, Inf) 1.000
50–59y	1.537 (0.418, 5.653) 0.517	1.703 (0.435, 6.664) 0.444	1.494 (0.218, 10.233) 0.683
60–69y	2.069 (0.607, 7.058) 0.246	2.284 (0.628, 8.308) 0.210	3.913 (0.697, 21.985) 0.121
70–80y	5.292 (1.139, 24.588) 0.034	4.401 (0.929, 20.847) 0.062	3.738 (0.679, 20.591) 0.130
Stratified by gender			
Men	3.507 (1.111, 11.066) 0.032	3.009 (0.904, 10.016) 0.073	4.557 (0.937, 22.147) 0.060
Women	2.744 (1.061, 7.093) 0.037	2.708 (1.025, 7.150) 0.044	2.673 (0.962, 7.426) 0.059
Stratified by race			
Hispanic	3.421 (0.884, 13.231) 0.075	3.109 (0.765, 12.625) 0.113	2.272 (0.329, 15.680) 0.405
Non-Hispanic White	1.978 (0.626, 6.248) 0.245	1.833 (0.570, 5.894) 0.309	1.688 (0.461, 6.179) 0.429
Non-Hispanic Black	4.560 (0.466, 44.664) 0.192	3.619 (0.360, 36.410) 0.275	0.157 (0.000, Inf) 1.000
Non-Hispanic Asian	6.300 (0.629, 63.127) 0.118	2.780 (0.228, 33.932) 0.423	Inf. (0.000, Inf) 1.000
Other races	2.667 (0.260, 27.382) 0.409	6.139 (0.461, 81.690) 0.169	Inf. (0.000, Inf) 1.000

*Model 1: Non-adjusted model; Model 2 adjusted for: gender; age; race; Model 3 adjusted for: gender; age; race; education; alcohol; diabetes; HBV infection; HCV infection; physical activity status; serum cotinine levels; BMI, and poverty income ratio*.

* *‘Inf' means that values can't be calculated*.

*NHANES, National Health and Nutrition Examination Survey; BMI, body mass index; HBV, hepatitis B virus; HCV, hepatitis C virus*.

In addition, subgroup analyses after PSM revealed that there was no statistically difference in demographic data including race, gender, and age.

## Discussion

The results showed that there was a positive correlation between cholecystectomy and LF or LC. In addition, the association remained statistically significant even after adjusting for possible confounders. Moreover, the association was still significant after exact PSM by age, gender, BMI, and diabetes.

Prior to this day, little research had been conducted on the correlation between cholecystectomy and LF or LC. Notably, a retrospective, multicenter study in Turkey showed no independent association between the presence of cholecystectomy and advanced LF (9). On the contrary, another cross-section study using data of the third US National Health and Nutrition Examination Survey (NHANES III, 1988–1994), showed the positive association of NAFLD with cholecystectomy (10). Nonetheless, research on the association between cholecystectomy and LF or LC is largely scarce. The results obtained herein were contrary to those reported in Turkey and may be a good update to understand the association of cholecystectomy and LF/LC (9). Cholecystectomy is the mainstream procedure for treating most gallbladder diseases and is associated with such complications as bile duct injury (0.08–0.5%), bile leak (0.42–1.1%), retained common bile duct stones (0.8–5.7%) and biliary strictures (0.4–0.6%) (26–30). These complications can in turn lead to prolonged hospital stays, increased morbidity, increased claims and more financial burden (30, 31). Moreover, obstruction of the bile duct caused by bile duct injury or biliary stricture may lead to LF, LC, and portal hypertension (32).

The possibility of correlation between cholecystectomy and LF or LC is further supported by the findings from the present study. After cholecystectomy, changes in bile flow and concentration of bile acid in the bile duct (33) might occur, which may cause chronic cholestasis, NAFLD and metabolic syndrome (10, 34–36). Interestingly, the study showed that participants with cholecystectomy for over 14 years had a higher incidence of LF than those <14 years (Supplementary Table 4).

Additionally, numerous studies have shown the positive association between metabolic syndrome and LF (25, 37). Moreover, the discovery of a bile acid shunt pathway between the gallbladder and liver, provided new insights on the protective role of the gallbladder (38). Interestingly, endocrine hormones secreted by the gallbladder, such as FGF19, may provide another possible mechanism for the development of metabolic syndrome after cholecystectomy (39–41).

These results highlighting the positive correlation between cholecystectomy and LF or LC in adults have an important implication in public health. Cholecystectomy is among the most common operations performed worldwide, with 750,000–1,000,000 procedures conducted in the United States, annually (42). Considering the early and delayed complications associated with cholecystectomy, it would be important to reassess the function and importance of the gallbladder (34). Strict surgical indications should also be implemented to reduce unnecessary cholecystectomy (43), given that preoperative evaluation of abdominal pain through gastroduodenoscopy was reported to be able to prevent 3.8% of cholecystectomies (44). Additionally, inexperienced surgeons should undergo standardized and strict training according to the operation protocols in order to reduce cholecystectomy-related bile duct injury (42). More importantly, annual monitoring of cholecystectomy patients should be conducted through liver ultrasound TE, especially those with such risk factors as being 40–59 years old, female, or Non-Hispanic White. This might help with the early diagnosis of LF or LC, hence enabling timely intervention (11). Moreover, further research is needed to identify the exact group of cholecystectomy patients who may be at a higher risk of developing LF or LC.

While the present study uncovered some insightful findings, it had a few limitations. First, the research results are not applicable to individuals younger than 20 years, including children and adolescents because of the age limit in cholecystectomy questionnaires used in NHANES 2017–2018. In addition, the study was not able to collect new data because this was a secondary analysis. Therefore, there might be a risk of residual confounding bias from the non-recorded covariates. Specifically, the results were not adjusted for cholecystectomy-related complications, which are potentially important contributors to LF or LC. Moreover, the study was unable to establish causality based on the cross-sectional data. On the other hand, it is ethically impossible to perform a randomized clinical trial on cholecystectomy in humans. Nonetheless, the study had several strengths, including a large sample size, a nationally representative population and use of exact PSM. As outcome variables, LF/LC were also assessed though the widely used TE in a standardized way, including repeated measurements to maintain accuracy.

In conclusion, the present study showed that cholecystectomy is positively associated with LF and LC in US adults, regardless of PSM. The discovery of this risk factors therefore provides new insights on the prevention of LF, LC.

## Data Availability Statement

Publicly available datasets were analyzed in this study. This data can be found here: https://wwwn.cdc.gov/nchs/nhanes/.

## Author Contributions

Z-QX and H-XL: contributed to the conception and design, the acquisition, analysis, interpretation of the data, and the drafting of the article or critical revision for important intellectual content. W-LT, LY, X-WM, W-XL, and Q-BW: collected data. C-ZS and Y-JC: contributed to the conception and design and the reviewing of the article or critical revision for important intellectual content. All authors approved the final version, and agree to be accountable for all aspects of the work.

## Funding

W-LT was supported by Grant 2020M683094 from China Postdoctoral Science Foundation. C-ZS was supported by Grant 82072714 from the National Natural Science Foundation of China. Y-JC was supported by Grant 81972263 from the National Natural Science Foundation of China and the program of Guangdong Provincial Clinical Research Center for Digestive Diseases (2020B1111170004).

## Conflict of Interest

The authors declare that the research was conducted in the absence of any commercial or financial relationships that could be construed as a potential conflict of interest.

## Publisher's Note

All claims expressed in this article are solely those of the authors and do not necessarily represent those of their affiliated organizations, or those of the publisher, the editors and the reviewers. Any product that may be evaluated in this article, or claim that may be made by its manufacturer, is not guaranteed or endorsed by the publisher.

## Abbreviations

ALT: alanine aminotransferase
AST: aspartate aminotransferase
ALP: alkaline Phosphatase
ALB: albumin
BMI: body mass index
CAP: controlled attenuation parameter
CI: confidence interval
GGT: gamma glutamyl transferase
HBV: hepatitis B virus
HCV: hepatitis C virus
IQR: interquartile range
LF: liver fibrosis
LC: liver cirrhosis
LSM: median liver stiffness
NAFLD: non-alcoholic fatty liver disease
NHANES: National Health and Nutrition Examination Survey
OR: odds ratio
PSM: propensity score matching
TC: total cholesterol
TB: total bilirubin.
