# Supplementary material for: Association of Cholecystectomy With Liver Fibrosis and Cirrhosis Among Adults in the USA: A Population-Based Propensity Score-Matched Study
Source: Front Med (Lausanne). 2021 Nov 30;8:787777. doi: 10.3389/fmed.2021.787777 (PMC8669563; doi:10.3389/fmed.2021.787777)
Supplement: Supplementary file 1 [file Data_Sheet_1.docx]

**Supplementary Material**

**1.Supplementary Results**

**1.1** **Overall Characteristics of All Participants**

For all participants, ***Supplemental Table 1*** showed that the incidence of Liver Fibrosis (LF) and Liver Cirrhosis (LC) was higher in participants who had received cholecystectomy [32.8% vs 19.8%, p<0.001; 5.0% vs 3.5%, p<0.001, respectively]. Moreover, participants who had LF or LC were more likely to have a history of cholecystectomy [18.8%(95%CI,16.5-21.1%) vs 9.8%(95%CI,8.8-10.8%), p<0.001; 17.3%(95%CI,11.0-23.6%) vs 11.5% (95%CI,10.6-12.4%), p=0.041, respectively] (***Supplemental Table 2 and Table 3***). Interestingly, ***Supplemental Table 4*** showed that the weighted mean time after cholecystectomy was 14.12±11.43 years, and participants with cholecystectomy for over 14 years had a higher incidence of LF than those less than 14 years [39.0%(95%CI,33.2-44.8%) vs 28.8%(95%CI,24.1-33.5%), P=0.0048], but not for LC (p=0.192).

**1.2 Associations between cholecystectomy and LF before propensity score matching (PSM)**

Next, the study assessed the association between cholecystectomy and LF (***Supplemental Table 5***) in participants before PSM. Analysis before adjusting for possible confounders and PSM showed that the OR value for the presence of LF in individuals who had undergone cholecystectomy, was 1.871[95%CI, 1.533-2.284], compared to those who had not received the surgery. Additionally, the value remained statistically significant after adjusting for gender, age, and race (OR, 1.997 [95%CI, 1.615-2.470]). However, there was a decrease in the OR value for the association between LF and cholecystectomy, after full adjustment (1.600 [95% CI, 1.278-2.002]).

In addition, subgroup analyses revealed that the OR value for the association of LF with cholecystectomy remained significant in participants who were 40-49 years old (Full adjustment: 2.323 [95% CI, 1.213-4.451]) and 50-59 years of age (Full adjustment: 2.601[95% CI, 1.500-4.511]). After stratification by gender, the OR value remained significant especially in females (Full adjustment: 1.675 [95% CI, 1.267-2.215]). Additionally, there were significant associations between cholecystectomy and other covariates, including non-Hispanic Whites (Full adjustment: OR,2.111 [95% CI,1.513-2.947]).

**1.3 Associations between cholecystectomy and LC before PSM**

Finally, the study assessed the association between cholecystectomy and LC (***Supplemental Table 6***) in participants before PSM. Analysis before adjusting for possible confounders and PSM showed that the OR value for the presence of LC in individuals with cholecystectomy, was 2.271 [95%CI, 1.490-3.460], compared to those who had not received the surgery. Additionally, the value remained statistically significant after adjusting for gender, age, and race (OR, 2.263 [95%CI, 1.441-3.553]). However, there was a decrease in the OR value for the association between LC and cholecystectomy, after full adjustment (1.733 [95% CI, 1.076-2.792]). Subgroup analyses revealed that cholecystectomy patients who are Non-Hispanic Asian (OR, 22.030 [95%CI,1.674-290.002]) are at a higher risk of developing LC.

**Supplemental Table 1** General characteristics of all participants(n=9254) by the presence or absence of a history of cholecystectomy in the NHANES 2017-2018

| Characters | Yes  (n=641) | No  (n=4925) | Not recorded  (n=3688) | *P* Value |
| --- | --- | --- | --- | --- |
| Age (years) | 57.64±15.21 | 47.01±17.19 | 9.75±5.95 | <0.001 |
| 0~9 | 0.0 | 0.0 | 48.6(47.0-50.2) |  |
| 10~19 | 0.0 | 0.0 | 51.3(49.7-52.9) |  |
| 20~29 | 4.7(3.1-6.3) | 20.3(19.2-21.4) | 0.0 |  |
| 30~39 | 10.3(7.9-12.7) | 18.8(17.1-19.9) | 0.0 |  |
| 40~49 | 17.4(14.5-20.3) | 16.0(15.0-17.0) | 0.0 |  |
| 50~59 | 17.4(14.5-20.3) | 18.7(17.6-19.8) | 0.0 |  |
| 60~69 | 25.6(22.2-29.0) | 14.0(13.0-15.0) | 0.0 |  |
| 70~80 | 24.6(21.3-27.9) | 12.2(11.3-13.1) | 0.0 |  |
| Gender |  |  |  | <0.001 |
| Male | 23.1(19.8-26.4) | 51.6(50.2-53.0) | 51.0(49.4-52.6) |  |
| Female | 76.9(73.6-80.2) | 48.4(47.0-49.8) | 49.0(47.4-50.6) |  |
| Race/ethnicity |  |  |  | <0.001 |
| Hispanic | 12.0(9.5-14.5) | 16.3(15.3-17.3) | 24.9(23.5-26.3) |  |
| Non-Hispanic White | 71.3(67.8-74.8) | 61.0(59.6-62.4) | 50.1(48.5-51.7) |  |
| Non-Hispanic Black | 7.2(5.2-9.2) | 12.0(11.1-12.9) | 13.0(11.9-14.1) |  |
| Non-Hispanic Asian | 2.3(1.1-3.5) | 6.4(5.7-7.1) | 4.7(4.0-5.4) |  |
| Other races^1^ | 7.2(5.2-9.2) | 4.3(3.7-4.9) | 7.3(6.5-8.1) |  |
| Education |  |  |  | <0.001 |
| More than high school | 57.4(53.6-61.2) | 62.0(60.6-63.4) | 2.6(2.1-3.1) |  |
| High school or equivalent | 30.6(27.0-34.2) | 26.6(25.4-27.8) | 5.7(5.0-6.4) |  |
| Less than high school | 11.7(9.2-14.2) | 11.3(10.4-12.2) | 63.0(61.4-64.6) |  |
| Not recorded | 0.2(-0.1,0.5) | 0.1(0.0-0.2) | 28.8(27.3-30.3) |  |
| Poverty-income ratio |  |  |  | <0.001 |
| <1.3 | 17.5(14.6-20.4) | 18.0(16.9-19.1) | 30.4(28.9-31.9) |  |
| 1.3-1.8 | 9.0 (6.8-11.2) | 8.4(7.6-9.2) | 10.0(9.0-11.0) |  |
| >1.8 | 63.7(60.0-67.4) | 62.6(61.2-64.0) | 50.2(48.6-51.8) |  |
| Not recorded | 9.8(7.5-12.1) | 10.9(10.0-11.8) | 9.4(8.5-10.3) |  |
| BMI group |  |  |  | <0.001 |
| <25 | 13.2(10.6-15.8) | 27.7(26.5-28.9) | 72.4(71.0-73.8) |  |
| 25–30 | 26.9(23.5-30.3) | 31.3(30.0-32.6) | 9.8(8.8-10.8) |  |
| ≥30 | 58.3(54.5-62.1) | 39.9(38.5-41.3) | 7.1(6.3-7.9) |  |
| Not recorded | 1.7(0.7-2.7) | 1.2(0.9-1.5) | 10.7(9.7-11.7) |  |
| Physical activity level |  |  |  | <0.001 |
| Inactive | 53.2(49.3-57.1) | 51.5(50.1-52.9) | 5.4(4.7-6.1) |  |
| Less active | 10.9(8.5-13.3) | 6.9(6.2-7.6) | 0.5(0.3-0.7) |  |
| Active | 35.9(32.2-39.6) | 41.6(40.2-43.0) | 4.4(3.7-5.1) |  |
| Not recorded | 0.0 | 0.0 | 89.7(88.7-90.7) |  |
| Daily alcohol drinking status |  |  |  | <0.001 |
| Non-drinkers | 6.9(4.9-8.9) | 7.0(6.3-7.7) | 2.6(2.1-3.1) |  |
| Moderate-drinkers | 28.9(25.4-32.4) | 29.9(28.6-31.2) | 1.7(1.3-2.1) |  |
| Heavy-drinkers | 16.9(14.0-19.8) | 13.4(12.4-14.4) | 2.0(1.5-2.5) |  |
| Binge-drinkers | 22.3(19.1-25.5) | 33.1(31.8-34.4) | 3.0(2.4-3.6) |  |
| Not recorded | 25.1(21.7-28.5) | 16.6(15.6-17.6) | 90.7(89.8-91.6) |  |
| History of diabetes |  |  |  | <0.001 |
| Yes | 24.6(21.3-27.9) | 12.3(11.4-13.2) | 0.7(0.4-1.0) |  |
| Having HBV infection |  |  |  | <0.001 |
| Yes | 0.9(0.2-1.6) | 0.9(0.6-1.2) | 40.9(39.3-42.5) |  |
| Having HCV infection |  |  |  | <0.001 |
| Yes | 2.5(1.3-3.7) | 2.4(2.0-2.8) | 42.0(40.4-43.6) |  |
| **Laboratory parameters** |  |  |  |  |
| Smoking(serum cotinine levels) |  |  |  | <0.001 |
| <0.015 | 44.1(40.3-47.9) | 35.4(34.1-36.7) | 25.2(23.8-26.6) |  |
| 0.015-3 | 31.4(27.8-35.0) | 36.0(34.7-37.3) | 38.7(37.1-40.3) |  |
| ≥3 | 21.8(18.6-25.0) | 23.6(22.4-24.8) | 5.0(4.3-5.7) |  |
| Not recorded | 2.7(1.4-4.0) | 5.1(4.5-5.7) | 31.0(29.5-32.5) |  |
| ALT(U/L) | 20.98± 15.73 | 23.25±17.13 | 16.91±16.99 | <0.001 |
| AST(U/L) | 20.47±11.83 | 22.44±13.52 | 20.44±10.42 | <0.001 |
| ALP(U/L) | 84.13±33.63 | 76.07±24.76 | 147.56± 97.84 | <0.001 |
| ALB(g/L) | 39.53±3.29 | 40.967±3.31 | 42.87±3.00 | <0.001 |
| GGT(U/L) | 31.18±60.90 | 29.83±36.19 | 14.78±9.68 | <0.001 |
| TC (mmol/L) | 4.81±0.97 | 4.90±1.06 | 4.05±0.72 | <0.001 |
| TB (umol/L) | 7.88±4.78 | 8.03±4.73 | 8.07±6.04 | 0.721 |
| Platelet(×10^9/L) | 256.00±72.94 | 243.19±60.61 | 284.01±68.58 | <0.001 |
| Transient Elastography |  |  |  |  |
| Median stiffness(kPa) | 6.94±6.87 | 5.77±5.20 | 5.01±2.91 | <0.001 |
| Controlled attenuated parameter(dB/m) | 278.07±62.44 | 262.27±62.91 | 219.84±53.59 | <0.001 |
| Liver fibrosis |  |  |  | <0.001 |
| Yes | 32.8(29.2-36.4) | 19.8(18.7-20.9) | 4.9(4.2-5.6) |  |
| Liver cirrhosis |  |  |  | <0.001 |
| Yes | 5.0(3.3-6.7) | 3.5(3.0-4.0) | 0.4(0.2-0.6) |  |

**Note:** Values are weighted mean±SD or weighted % (95% confidence interval). P values are weighted. ^1^Other races include American Indian or Alaska Native, Native Hawaiian or other Pacific Islander, and multiracial persons.

**Abbreviations:** NHANES, National Health and Nutrition Examination Survey; BMI, body mass index; HBV, hepatitis B virus; HCV, hepatitis C virus; ALT, alanine aminotransferase; AST, aspartate aminotransferase; ALP, alkaline Phosphatase; ALB, albumin; GGT, gamma glutamyl transferase; TC, total cholesterol; TB, total bilirubin.

**Supplemental Table 2** General characteristics of included participants(n=4497) by the presence or absence of significant liver fibrosis in the NHANES 2017-2018

| Characters | Yes  (n=1085) | No  (n=3412) | *p*-Value |
| --- | --- | --- | --- |
| Age (years) | 51.34±16.80 | 46.98±16.98 | <0.001 |
| 20~29 | 13.4(11.4-15.4) | 20.1(18.8-21.4) |  |
| 30~39 | 15.5(13.3-17.7) | 18.8(17.5-20.1) |  |
| 40~49 | 13.7(11.7-15.7) | 16.3(15.1-17.5) |  |
| 50~59 | 21.2(18.8-23.6) | 18.6(17.3-19.9) |  |
| 60~69 | 20.5(18.1-22.9) | 14.9(13.7-16.1) |  |
| 70~80 | 15.8(13.6-18.0) | 11.2(10.1-12.3) |  |
| Gender |  |  | <0.001 |
| Male | 58.3(55.4-61.2) | 46.9(45.2-48.6) |  |
| Female | 41.7(38.8-44.6) | 53.1(51.4-54.8) |  |
| Race/ethnicity |  |  | 0.115 |
| Hispanic | 15.5(13.3-17.7) | 15.8(14.6-17.0) |  |
| Non-Hispanic White | 62.4(59.5-65.3) | 62.5(60.9-64.1) |  |
| Non-Hispanic Black | 12.7(10.7-14.7) | 10.8(9.8-11.8) |  |
| Non-Hispanic Asian | 4.3(3.1-5.5) | 6.2(5.4-7.0) |  |
| Other races^1^ | 5.0(3.7-6.3) | 4.6(3.9-5.3) |  |
| Education level |  |  | <0.001 |
| More than high school | 55.7(52.7-58.7) | 63.9(62.3-65.5) |  |
| High school or equivalent | 30.9(28.2-33.6) | 25.7(24.2-27.2) |  |
| Less than high school | 13.4(11.4-15.4) | 10.4(9.4-11.4) |  |
| Not recorded | 0.04(-0.1-0.2) | 0.1(0.0-0.2) |  |
| Poverty-income ratio |  |  | 0.328 |
| <1.3 | 18.6(16.3-20.9) | 17.3(16.0-18.6) |  |
| 1.3-1.8 | 9.1(7.4-10.8) | 8.0(7.1-8.9) |  |
| >1.8 | 63.3(60.4-66.2) | 64.2(62.6-65.8) |  |
| Not recorded | 9.0(7.3-10.7) | 10.5(9.5-11.5) |  |
| BMI group |  |  | <0.001 |
| <25 | 15.7(13.5-17.9) | 30.3(28.8-31.8) |  |
| 25–30 | 20.1(17.7-22.5) | 34.4(32.8-36.0) |  |
| ≥30 | 63.3(60.4-66.2) | 34.9(33.3-36.5) |  |
| Not recorded | 0.9(0.3-1.5) | 0.5(0.3-0.7) |  |
| Physical activity level |  |  | <0.001 |
| Inactive | 46.7(43.7-49.7) | 52.0(50.3-53.7) |  |
| Less active | 10.1(8.3-11.9) | 7.1(6.2-8.0) |  |
| Active | 43.3(40.4-46.2) | 40.9(39.3-42.5) |  |
| Daily alcohol drinking status |  |  | 0.198 |
| Non-drinkers | 6.5(5.0-8.0) | 7.3(6.4-8.2) |  |
| Moderate-drinkers | 27.4(24.7-30.1) | 30.3(28.8-31.8) |  |
| Heavy-drinkers | 14.3(12.2-16.4) | 14.0(12.8-15.2) |  |
| Binge-drinkers | 33.9(31.1-36.7) | 32.7(31.1-34.3) |  |
| Not recorded | 18.0(15.7-20.3) | 15.6(14.4-16.8) |  |
| History of diabetes |  |  | <0.001 |
| Yes | 27.9(25.2-30.6) | 8.7(7.8-9.6) |  |
| Having HBV infection |  |  | 0.025 |
| Yes | 0.8(0.3-1.3) | 0.9(0.6-1.2) |  |
| Having HCV infection |  |  | <0.001 |
| Yes | 5.5(4.1-6.9) | 1.6(1.2-2.0) |  |
| History of cholecystectomy |  |  | <0.001 |
| Yes | 18.8(16.5-21.1) | 9.8(8.8-10.8) |  |
| **Laboratory parameters** |  |  |  |
| Smoking(serum cotinine levels) |  |  | 0.066 |
| <0.015 | 36.1(33.2-39.0) | 37.2(35.6-38.8) |  |
| 0.015-3 | 34.3(31.5-37.1) | 36.3(34.7-37.9) |  |
| ≥3 | 24.6(22.0-27.2) | 23.1(21.7-24.5) |  |
| Not recorded | 5.0(3.7-6.3) | 3.4(2.8-4.0) |  |
| ALT(U/L) | 31.40±25.63 | 21.20±13.42 | <0.001 |
| AST(U/L) | 27.22±21.00 | 20.97±9.88 | <0.001 |
| ALP(U/L) | 82.31±32.47 | 74.55± 22.46 | <0.001 |
| ALB(g/L) | 40.32±3.27 | 41.15±3.14 | <0.001 |
| GGT(U/L) | 46.17±66.95 | 25.72±27.74 | <0.001 |
| TC(mmol/L) | 4.79±1.05 | 4.94±1.03 | <0.001 |
| TB(umol/L) | 8.04±4.41 | 8.10±4.82 | 0.761 |
| Platelet(×10^9/L) | 240.85±65.52 | 245.77±60.03 | 0.030 |

**Note:** Values are weighted mean±SD or weighted % (95% confidence interval). P values are weighted. ^1^Other races include American Indian or Alaska Native, Native Hawaiian or other Pacific Islander, and multiracial persons.

**Abbreviations:** NHANES, National Health and Nutrition Examination Survey; BMI, body mass index; HBV, hepatitis B virus; HCV, hepatitis C virus; ALT, alanine aminotransferase; AST, aspartate aminotransferase; ALP, alkaline Phosphatase; ALB, albumin; GGT, gamma glutamyl transferase; TC, total cholesterol; TB, total bilirubin.

**Supplemental Table 3** General characteristics of included participants(n=4497) by the presence or absence of liver cirrhosis in the NHANES 2017-2018

| Characters | Yes  (n=137) | No  (n=4360) | *p*-Value |
| --- | --- | --- | --- |
| Age (years) | 53.68±14.72 | 47.74±17.07 | <0.001 |
| 20~29 | 5.2(1.5-8.9) | 19.1(17.9-20.3) |  |
| 30~39 | 18.3(11.8-24.8) | 18.1(17.0-19.2) |  |
| 40~49 | 7.9(3.4-12.4) | 16.0(14.9-17.1) |  |
| 50~59 | 31.1(23.3-38.9) | 18.8(17.6-20.0) |  |
| 60~69 | 23.8(16.7-30.9) | 15.9(14.8-17.0) |  |
| 70~80 | 13.7(7.9-19.5) | 12.2(11.2-13.2) |  |
| Gender |  |  | <0.001 |
| Male | 64.3(56.3-72.3) | 48.9(47.4-50.4) |  |
| Female | 35.7(27.7-43.7) | 51.1(49.6-52.6) |  |
| Race/ethnicity |  |  | 0.107 |
| Hispanic | 14.4(8.5-20.3) | 15.8(14.7-16.9) |  |
| Non-Hispanic White | 69.7(62.0-77.4) | 62.3(60.9-63.7) |  |
| Non-Hispanic Black | 6.0(2.0-10.0) | 11.4(10.5-12.3) |  |
| Non-Hispanic Asian | 3.2(0.3-6.1) | 5.9(5.2-6.6) |  |
| Other races^1^ | 6.8(2.6-11.0) | 4.7(4.1-5.3) |  |
| Education |  |  | <0.001 |
| More than high school | 38.6(30.4) | 62.9(61.5-64.3) |  |
| High school or equivalent | 49.0(46.8) | 26.1(24.8-27.4) |  |
| Less than high school | 12.3(6.8-17.8) | 11.0(10.1-11.9) |  |
| Not recorded | 0.0 | 0.1(0.0-0.2) |  |
| Poverty-income ratio |  |  | 0.342 |
| <1.3 | 18.1(11.7-24.5) | 17.6(16.5-18.7) |  |
| 1.3-1.8 | 11.7(6.3-17.1) | 8.1(7.3-8.9) |  |
| >1.8 | 63.2(55.1-71.3) | 64.0(62.6-65.4) |  |
| Not recorded | 7.1(2.8-11.4) | 10.3(9.4-11.2) |  |
| BMI group |  |  | <0.001 |
| <25 | 11.7(6.3-17.1) | 27.6(26.3-28.9) |  |
| 25–30 | 13.5(7.8-19.2) | 31.8(30.4-33.2) |  |
| ≥30 | 74.1(66.8-81.4) | 40.0(38.5-41.5) |  |
| Not recorded | 0.6(-0.7,1.9) | 0.6(0.4-0.8) |  |
| Physical activity level |  |  | 0.028 |
| Inactive | 40.7(32.5-48.9) | 51.2(49.7-52.7) |  |
| Less active | 6.8(2.6-11.0) | 7.7(6.9-8.5) |  |
| Active | 52.5(44.1-60.9) | 41.1(39.6-42.6) |  |
| Daily alcohol drinking status |  |  | 0.300 |
| Non-drinkers | 9.7(4.7-14.7) | 7.1(6.3-7.9) |  |
| Moderate-drinkers | 25.1(17.8-32.4) | 29.9(28.5-31.3) |  |
| Heavy-drinkers | 12.3(6.8-17.8) | 14.2(13.2-15.2) |  |
| Binge-drinkers | 39.3(31.1-47.5) | 32.7(31.3-34.1) |  |
| Not recorded | 13.6(7.9-19.3) | 16.2(15.1-17.3) |  |
| History of diabetes |  |  | <0.001 |
| Yes | 38.7(30.5-46.9) | 12.1(11.1-13.1) |  |
| Having HBV infection |  |  | 0.245 |
| Yes | 0.2(-0.5,0.9) | 0.9(0.6-1.2) |  |
| Having HCV infection |  |  | <0.001 |
| Yes | 16.2(10.0-22.4) | 2.0(1.6-2.4) |  |
| History of cholecystectomy |  |  | 0.041 |
| Yes | 17.3(11.0-23.6) | 11.5(10.6-12.4) |  |
| **Laboratory parameters** |  |  |  |
| Smoking(serum cotinine levels) |  |  | 0.007 |
| <0.015 | 42.6(34.3-50.9) | 36.8(35.4-38.2) |  |
| 0.015-3 | 24.7(17.5-31.9) | 36.2(34.8-37.6) |  |
| ≥3 | 31.4(23.6-39.2) | 23.2(21.9-24.5) |  |
| Not recorded | 1.3(-0.6,3.2) | 3.8(3.2-4.4) |  |
| ALT(U/L) | 45.60±39.62 | 22.66±15.56 | <0.001 |
| AST(U/L) | 40.36±35.64 | 21.72±11.46 | <0.001 |
| ALP(U/L) | 88.55±35.34 | 75.81±24.63 | <0.001 |
| ALB(g/L) | 39.57±3.89 | 41.02±3.15 | <0.001 |
| GGT(U/L) | 68.16±81.73 | 28.86±37.69 | <0.001 |
| TC(mmol/L) | 4.80±1.14 | 4.91±1.03 | 0.263 |
| TB(umol/L) | 9.61± 5.10 | 8.04±4.71 | <0.001 |
| Platelet(×10^9/L) | 220.04±65.90 | 245.51±60.96 | <0.001 |

**Note:** Values are weighted mean±SD or weighted % (95% confidence interval). P values are weighted. ^1^Other races include American Indian or Alaska Native, Native Hawaiian or other Pacific Islander, and multiracial persons.

**Abbreviations:** NHANES, National Health and Nutrition Examination Survey; BMI, body mass index; HBV, hepatitis B virus; HCV, hepatitis C virus; ALT, alanine aminotransferase; AST, aspartate aminotransferase; ALP, alkaline Phosphatase; ALB, albumin; GGT, gamma glutamyl transferase; TC, total cholesterol; TB, total bilirubin.

**Supplemental Table 4** General characteristics of participants(n=641) by mean time after cholecystectomy in the NHANES 2017-2018

| Characters | Total  (n=641) | Postoperative Time≤14years  (n=356) | Postoperative Time>14years  (n=274) | Not recorded  (n=11) | *p*-Value |
| --- | --- | --- | --- | --- | --- |
| Mean time after cholecystectomy(years) | 14.12±11.43 | 6.47±4.20 | 25.52±9.04 | - | <0.001 |
| Age (years) | 57.64± 15.21 | 53.38±15.82 | 63.65± 11.73 | 75.11± 6.60 | <0.001 |
| 20~29 | 4.7(3.1-6.3) | 8.0(5.2-10.8) | 0.0 | 0.0 |  |
| 30~39 | 10.3(7.9-12.7) | 14.3(10.7-17.9) | 4.4(2.0-6.8) | 0.0 |  |
| 40~49 | 17.4(14.5-20.3) | 24.0(19.6-28.4) | 7.8(4.6-11.0) | 0.0 |  |
| 50~59 | 17.4(14.5-20.3) | 15.8(12.0-19.6) | 20.1(15.4-24.8) | 0.0 |  |
| 60~69 | 25.6(22.2-29.0) | 18.8(14.7-22.9) | 35.9(30.2-41.6) | 23.8(-1.4,49.0) |  |
| 70~80 | 24.6(21.3-27.9) | 19.1(15.0-23.2) | 31.8(26.3-37.3) | 76.2(51.0-101.4) |  |
| Gender |  |  |  |  | 0.014 |
| Male | 23.1(19.8-26.4) | 26.9(22.3-31.5) | 17.2(12.7-21.7) | 33.1(5.3-60.9) |  |
| Female | 76.9(73.6-80.2) | 73.1(68.5-77.7) | 82.8(78.3-87.3) | 66.9(39.1-94.7) |  |
| Race/ethnicity |  |  |  |  | 0.302 |
| Hispanic | 12.0(9.5-14.5) | 13.4(9.9-16.9) | 10.3(6.7-13.9) | 0.0 |  |
| Non-Hispanic White | 71.3(67.8-74.8) | 70.7(66.0-75.4) | 72.4(67.1-77.7) | 66.4(38.5-94.3) |  |
| Non-Hispanic Black | 7.2(5.2-9.2) | 7.6(4.8-10.4) | 6.3(3.4-9.2) | 21.9(-2.5,46.3) |  |
| Non-Hispanic Asian | 2.3(1.1-3.5) | 2.6(0.9-4.3) | 1.6(0.1-3.1) | 11.7(-7.3,30.7) |  |
| Other races^1^ | 7.2(5.2-9.2) | 5.8(3.4-8.2) | 9.4(5.9-12.9) | 0.0 |  |
| Education |  |  |  |  | <0.001 |
| More than high school | 57.4(53.6-61.2) | 57.4(52.3-62.5) | 57.6(51.7-63.5) | 48.5(19.0-78.0) |  |
| High school or equivalent | 30.6(27.0-34.2) | 34.2(29.3-39.1) | 25.6(20.4-30.8) | 15.8(-5.8,37.4) |  |
| Less than high school | 11.7(9.2-14.2) | 8.4(5.5-11.3) | 16.8(12.4-21.2) | 5.8(-0.8,19.6) |  |
| Not recorded | 0.2(-0.1,0.5) | 0.0 | 0.0 | 29.9(2.8-57.0) |  |
| Poverty-income ratio |  |  |  |  | 0.050 |
| <1.3 | 17.5(14.6-20.4) | 20.0(15.8-24.2) | 13.6(9.5-17.7) | 21.6(-2.7,45.9) |  |
| 1.3-1.8 | 9.0(6.8-11.2) | 6.9(4.3-9.5) | 12.1(8.2-16.0) | 8.0(-8.0,24.0) |  |
| >1.8 | 63.7(60.0-67.4) | 63.1(58.1-68.1) | 65.2(59.6-70.8) | 34.3(6.2-62.4) |  |
| Not recorded | 9.8(7.5-12.1) | 10.0(6.9-13.1) | 9.1(5.7-12.5) | 36.1(7.7-64.5) |  |
| BMI group |  |  |  |  | 0.162 |
| <25 | 13.2(10.6-15.8) | 11.3(8.0-14.6) | 16.3(11.9-20.7) | 0.0 |  |
| 25–30 | 26.9(23.5-30.3) | 27.6(23.0-32.2) | 24.9(19.8-30.0) | 74.1(48.2-100.0) |  |
| ≥30 | 58.3(54.5-62.1) | 59.5(54.4-64.6) | 57.0(51.1-62.9) | 25.9(0.0-51.8) |  |
| Not recorded | 1.7(0.7-2.7) | 1.6(0.3-2.9) | 1.8(0.2-3.4) | 0.0 |  |
| Physical activity level |  |  |  |  | 0.003 |
| Inactive | 53.2(49.3-57.1) | 47.7(42.5-52.9) | 60.8(55.0-66.6) | 85.0(63.9-106.1) |  |
| Less active | 10.9(8.5-13.3) | 10.6(7.4-13.8) | 11.2(7.5-14.9) | 15.0(-6.1,36.1) |  |
| Active | 35.9(32.3-39.6) | 41.7(36.6-46.8) | 28.0(22.7-33.3) | 0.0 |  |
| Daily alcohol drinking status |  |  |  |  | <0.001 |
| Non-drinkers | 6.9(4.9-8.9) | 4.3(2.2-6.4) | 10.3(6.7-13.9) | 32.9(5.1-60.7) |  |
| Moderate-drinkers | 28.9(25.4-32.4) | 30.0(25.2-34.8) | 27.9(22.6-33.2) | 0.0 |  |
| Heavy-drinkers | 16.9(14.0-19.8) | 16.9(13.0-20.8) | 16.9(12.5-21.3) | 8.0(-8.0,24.0) |  |
| Binge-drinkers | 22.3(19.1-25.5) | 27.6(23.0-32.2) | 14.7(10.5-18.9) | 0.0 |  |
| Not recorded | 25.1(21.7-28.5) | 21.1(16.9-25.3) | 30.2(24.8-35.6) | 59.1(30.0-88.2) |  |
| History of diabetes |  |  |  |  | 0.001 |
| Yes | 24.6(21.3-27.9) | 18.6(14.6-22.6) | 33.5(27.9-39.1) | 31.7(4.2-59.2) |  |
| Having HBV infection |  |  |  |  | 0.289 |
| Yes | 0.9(0.2-1.6) | 0.2(-0.3,0.7) | 1.8(0.2-3.4) | 0.0 |  |
| Having HCV infection |  |  |  |  | 0.833 |
| Yes | 2.5(1.3-3.7) | 3.1(1.3-4.9) | 1.7(0.2-3.2) | 0.0 |  |
| **Laboratory parameters** |  |  |  |  |  |
| Smoking(serum cotinine levels) |  |  |  |  | 0.255 |
| <0.015 | 44.1(40.3-47.9) | 40.3(35.2-45.4) | 49.4(43.5-55.3) | 58.8(29.7-87.9) |  |
| 0.015-3 | 31.4(27.8-35.0) | 31.9(27.1-36.7) | 30.7(25.2-36.2) | 25.9(0.0-51.8) |  |
| ≥3 | 21.8(18.6-25.0) | 25.1(20.6-29.6) | 17.2(12.7-21.7) | 15.3(-6.0,36.6) |  |
| Not recorded | 2.7(1.4-4.0) | 2.7(1.0-4.4) | 2.8(0.8-4.8) | 0.0 |  |
| ALT(U/L) | 20.98± 15.73 | 22.22±17.40 | 19.22± 12.81 | 17.93± 7.36 | 0.077 |
| AST(U/L) | 20.47± 11.83 | 20.86±12.07 | 19.90± 11.55 | 20.64± 2.80 | 0.637 |
| ALP(U/L) | 84.13± 33.63 | 85.31±38.68 | 82.57± 24.47 | 73.36± 21.80 | 0.510 |
| ALB(g/L) | 39.53± 3.29 | 39.50±3.21 | 39.57± 3.40 | 39.55± 3.92 | 0.969 |
| GGT(U/L) | 31.18± 60.89 | 36.07±76.61 | 24.12± 22.47 | 23.60± 15.37 | 0.069 |
| TC(mmol/L) | 4.81± 0.97 | 4.84±0.90 | 4.78±1.05 | 4.48± 1.11 | 0.568 |
| TB(umol/L) | 7.88± 4.78 | 8.05±5.07 | 7.55±4.17 | 11.05± 8.20 | 0.183 |
| Platelet(×10^9/L) | 256.00 ± 72.94 | 260.70± 75.49 | 250.24± 68.37 | 192.55± 45.45 | 0.041 |
| **Transient Elastography** |  |  |  |  |  |
| Median stiffness(kPa) | 6.94± 6.87 | 6.89±7.23 | 7.03±6.32 | 6.25± 3.70 | 0.957 |
| Controlled attenuated parameter(dB/m) | 278.07± 62.44 | 276.07± 65.87 | 280.88± 56.63 | 297.01± 55.55 | 0.593 |
| **Liver fibrosis** |  |  |  |  | 0.0048 |
| Yes | 32.8(29.2-36.4) | 28.8(24.1-33.5) | 39.0(33.2-44.8) | 15.6(-5.8,37.0) |  |
| **Liver cirrhosis** |  |  |  |  | 0.192 |
| Yes | 5.0(3.3-6.7) | 5.7(3.3-8.1) | 3.9(1.6-6.2) | 7.6(-8.1,23.3) |  |

**Note:** Values are weighted mean±SD or weighted % (95% confidence interval). P values are weighted.  ^1^Other races include American Indian or Alaska Native, Native Hawaiian or other Pacific Islander, and multiracial persons.

**Abbreviations:** NHANES, National Health and Nutrition Examination Survey; BMI, body mass index; HBV, hepatitis B virus; HCV, hepatitis C virus; ALT, alanine aminotransferase; AST, aspartate aminotransferase; ALP, alkaline Phosphatase; ALB, albumin; GGT, gamma glutamyl transferase; TC, total cholesterol; TB, total bilirubin.

**Supplemental Table 5** Associations between cholecystectomy and significant liver fibrosis for included participants(n=4497), NHANES 2017–2018.

|  | Model1 OR (95%CI), P | Model2 OR (95%CI), P | Model3 OR (95%CI), P |
| --- | --- | --- | --- |
| Cholecystectomy |  |  |  |
| No | Reference | Reference | Reference |
| Yes | 1.871 (1.533, 2.284) <0.001 | 1.997 (1.615, 2.470) <0.001 | 1.600 (1.278, 2.002) <0.001 |
| Stratified by Age |  |  |  |
| 20~29y | 1.501 (0.542, 4.157) 0.434 | 1.789 (0.632, 5.062) 0.273 | 1.629 (0.545, 4.864)  0.382 |
| 30~39y | 2.625 (1.349, 5.107) 0.004 | 2.896 (1.430, 5.866) 0.003 | 2.094 (0.975, 4.498)  0.058 |
| 40~49y | 3.133 (1.849, 5.309) <0.001 | 4.126 (2.323, 7.326) <0.001 | 2.323 (1.213, 4.451)  0.011 |
| 50~59y | 2.589 (1.638, 4.090) <0.001 | 3.117 (1.923, 5.053) <0.001 | 2.601 (1.500, 4.511)  0.001 |
| 60~69y | 0.981 (0.651, 1.477) 0.926 | 1.176 (0.766, 1.804) 0.458 | 0.973 (0.611, 1.547)  0.907 |
| 70~80y | 1.373 (0.931, 2.025) 0.109 | 1.491 (0.996, 2.232) 0.052 | 1.260 (0.821, 1.935)  0.290 |
| Stratified by gender |  |  |  |
| Male | 1.871 (1.300, 2.700) 0.001 | 1.691 (1.160, 2.465) 0.006 | 1.353 (0.908, 2.017) 0.137 |
| Female | 2.388 (1.863, 3.061) <0.001 | 2.166 (1.672, 2.807) <0.001 | 1.675 (1.267, 2.215) <0.001 |
| Stratified by race |  |  |  |
| Hispanic | 1.787 (1.172, 2.723) 0.007 | 1.674 (1.064, 2.635) 0.026 | 1.302 (0.790, 2.146) 0.300 |
| Non-Hispanic White | 2.231 (1.665, 2.989) <0.001 | 2.560 (1.873, 3.498) <0.001 | 2.111 (1.513, 2.947) <0.001 |
| Non-Hispanic Black | 0.927 (0.547, 1.571) 0.778 | 0.942 (0.545, 1.630) 0.832 | 0.958 (0.542, 1.693) 0.882 |
| Non-Hispanic Asian | 2.668 (1.254, 5.679) 0.011 | 2.458 (1.127, 5.358) 0.024 | 1.514 (0.587, 3.906) 0.391 |
| Other races | 2.280 (1.038, 5.004) 0.040 | 3.049 (1.290, 7.203) 0.011 | 2.646 (0.970, 7.219) 0.057 |

**Note:** Model 1: Non-adjusted model; Model 2 adjusted for: gender; age; race; Model 3 adjusted for: gender; age; race; education; alcohol; diabetes; HBV infection; HCV infection; physical activity status; serum cotinine levels; BMI, and poverty income ratio.

**Abbreviations:** NHANES, National Health and Nutrition Examination Survey; BMI, body mass index; HBV, hepatitis B virus; HCV, hepatitis C virus.

**Supplemental Table 6** Associations between cholecystectomy and liver cirrhosis for included participants(n=4497), NHANES 2017–2018.

|  | Model1 OR (95%CI), P | Model2 OR (95%CI), P | Model3 OR (95%CI), P |
| --- | --- | --- | --- |
| Cholecystectomy |  |  |  |
| No | Reference | Reference | Reference |
| Yes | 2.271 (1.490, 3.460) <0.001 | 2.263 (1.441, 3.553) <0.001 | 1.733 (1.076, 2.792) 0.024 |
| Stratified by Age |  |  |  |
| 20~29y | 0.000 (0.000, Inf)*  0.992 | 0.000 (0.000, Inf) 0.998 | 0.000 (0.000, Inf)  0.999 |
| 30~39y | 4.303 (1.165, 15.894) 0.029 | 3.328 (0.819, 13.518) 0.093 | 2.058 (0.336, 12.600) 0.435 |
| 40~49y | 3.459 (0.658, 18.172) 0.143 | 6.824 (1.055, 44.152) 0.044 | Inf. (0.000, Inf)  0.996 |
| 50~59y | 1.989 (0.792, 4.995) 0.143 | 2.409 (0.895, 6.480) 0.082 | 1.998 (0.615, 6.492) 0.249 |
| 60~69y | 1.279 (0.586, 2.793) 0.537 | 1.681 (0.737, 3.831) 0.21688 | 1.362 (0.565, 3.280) 0.491 |
| 70~80y | 2.328 (1.051, 5.155) 0.037 | 2.102 (0.923, 4.785) 0.077 | 1.533 (0.624, 3.766) 0.352 |
| Stratified by gender |  |  |  |
| Male | 3.038 (1.638, 5.636) <0.001 | 2.408 (1.264, 4.589) 0.008 | 1.960 (0.980, 3.917) 0.057 |
| Female | 2.700 (1.466, 4.974) 0.001 | 2.119 (1.125, 3.991) 0.020 | 1.648 (0.841, 3.231) 0.146 |
| Stratified by race |  |  |  |
| Hispanic | 2.413 (1.077, 5.409) 0.032 | 1.841 (0.767, 4.419) 0.172 | 1.211 (0.449, 3.265) 0.706 |
| Non-Hispanic White | 1.464 (0.762, 2.815) 0.253 | 1.607 (0.811, 3.185) 0.174 | 1.157 (0.560, 2.392) 0.694 |
| Non-Hispanic Black | 2.116 (0.609, 7.345) 0.238 | 2.373 (0.613, 9.182) 0.211 | 3.207 (0.652, 15.784) 0.152 |
| Non-Hispanic Asian | 6.800 (1.750, 26.417) 0.006 | 6.214 (1.464, 26.382) 0.013 | 22.030 (1.674, 290.002) 0.019 |
| Other races | 3.238 (0.790, 13.274) 0.102 | 6.650 (1.284, 34.447) 0.024 | 9.375 (0.440, 199.802) 0.152 |

**Note:** Model 1: Non-adjusted model; Model 2 adjusted for: gender; age; race; Model 3 adjusted for: gender; age; race; education; alcohol; diabetes; HBV infection; HCV infection; physical activity status; serum cotinine levels; BMI, and poverty income ratio. *’Inf’ means these values can't be calculated.

**Abbreviations:** NHANES, National Health and Nutrition Examination Survey; BMI, body mass index; HBV, hepatitis B virus; HCV, hepatitis C virus.
